# Supplementary material for: Resolving the full spectrum of human genome variation using Linked-Reads
Source: Genome Res. 2019 Apr;29(4):635–45. doi: 10.1101/gr.234443.118 (PMC6442396; doi:10.1101/gr.234443.118)
Supplement: Supplemental Material [file supp_gr.234443.118_Supplemental_longranger-2.2.zip › longranger-2.2.tar.bz2!/lr2.2-public/lib/stan/cmdstan/stan/doc/api/html/statement__grammar__def_8hpp_source.html]

Stan: src/stan/gm/grammars/statement\_grammar\_def.hpp Source File


|  |  |
| --- | --- |
|  | Stan  2.5.0  probability, sampling & optimization |


- Main Page
- Related Pages
- Namespaces
- Classes
- Files

- File List
- File Members

All Classes Namespaces Files Functions Variables Typedefs Enumerations Enumerator Friends Macros Pages

- src
- stan
- gm
- grammars

statement\_grammar\_def.hpp

Go to the documentation of this file.

1 #ifndef STAN\_\_GM\_\_PARSER\_\_STATEMENT\_GRAMMAR\_DEF\_\_HPP

2 #define STAN\_\_GM\_\_PARSER\_\_STATEMENT\_GRAMMAR\_DEF\_\_HPP

3

4 #include <cstddef>

5 #include <iomanip>

6 #include <iostream>

7 #include <istream>

8 #include <map>

9 #include <set>

10 #include <sstream>

11 #include <string>

12 #include <utility>

13 #include <vector>

14 #include <stdexcept>

15

16

17 #include <boost/spirit/include/qi.hpp>

18 #include <boost/lexical\_cast.hpp>

19 #include <boost/fusion/include/adapt\_struct.hpp>

20 #include <boost/fusion/include/std\_pair.hpp>

21 #include <boost/config/warning\_disable.hpp>

22 #include <boost/spirit/include/qi\_numeric.hpp>

23 #include <boost/spirit/include/phoenix\_core.hpp>

24 #include <boost/spirit/include/phoenix\_function.hpp>

25 #include <boost/spirit/include/phoenix\_fusion.hpp>

26 #include <boost/spirit/include/phoenix\_object.hpp>

27 #include <boost/spirit/include/phoenix\_operator.hpp>

28 #include <boost/spirit/include/phoenix\_stl.hpp>

29 #include <boost/spirit/include/support\_multi\_pass.hpp>

30 #include <boost/tuple/tuple.hpp>

31 #include <boost/variant/apply\_visitor.hpp>

32 #include <boost/variant/recursive\_variant.hpp>

33

34 #include <boost/spirit/include/version.hpp>

35 #include <boost/spirit/include/support\_line\_pos\_iterator.hpp>

36

37 #include <stan/gm/ast.hpp>

38 #include <stan/gm/grammars/whitespace\_grammar.hpp>

39 #include <stan/gm/grammars/expression\_grammar.hpp>

40 #include <stan/gm/grammars/var\_decls\_grammar.hpp>

41 #include <stan/gm/grammars/statement\_grammar.hpp>

42 #include <stan/gm/grammars/common\_adaptors\_def.hpp>

43

44 BOOST\_FUSION\_ADAPT\_STRUCT(stan::gm::assignment,

45  (stan::gm::variable\_dims, var\_dims\_)

46  (stan::gm::expression, expr\_) );

47

48 BOOST\_FUSION\_ADAPT\_STRUCT(stan::gm::variable\_dims,

49  (std::string, name\_)

50  (std::vector<stan::gm::expression>, dims\_) );

51

52 BOOST\_FUSION\_ADAPT\_STRUCT(stan::gm::distribution,

53  (std::string, family\_)

54  (std::vector<stan::gm::expression>, args\_) );

55

56 BOOST\_FUSION\_ADAPT\_STRUCT(stan::gm::for\_statement,

57  (std::string, variable\_)

58  (stan::gm::range, range\_)

59  (stan::gm::statement, statement\_) );

60

61 BOOST\_FUSION\_ADAPT\_STRUCT(stan::gm::return\_statement,

62  (stan::gm::expression, return\_value\_) );

63

64 BOOST\_FUSION\_ADAPT\_STRUCT(stan::gm::print\_statement,

65  (std::vector<stan::gm::printable>, printables\_) );

66

67 BOOST\_FUSION\_ADAPT\_STRUCT(stan::gm::reject\_statement,

68  (std::vector<stan::gm::printable>, printables\_) );

69

70 BOOST\_FUSION\_ADAPT\_STRUCT(stan::gm::increment\_log\_prob\_statement,

71  (stan::gm::expression, log\_prob\_) );

72

73 BOOST\_FUSION\_ADAPT\_STRUCT(stan::gm::sample,

74  (stan::gm::expression, expr\_)

75  (stan::gm::distribution, dist\_)

76  (stan::gm::range, truncation\_) );

77

78 BOOST\_FUSION\_ADAPT\_STRUCT(stan::gm::statements,

79  (std::vector<stan::gm::var\_decl>, local\_decl\_)

80  (std::vector<stan::gm::statement>, statements\_) );

81

82 namespace stan {

83

84  namespace gm {

85

86  struct validate\_return\_allowed {

87  template <typename T1, typename T2, typename T3>

88  struct result { typedef void type; };

89  void operator()(var\_origin origin,

90  bool& pass,

91  std::ostream& error\_msgs) const {

92  if (origin != function\_argument\_origin

93  && origin != function\_argument\_origin\_lp

94  && origin != function\_argument\_origin\_rng) {

95  error\_msgs << "Returns only allowed from function bodies." << std::endl;

96  pass = false;

97  return;

98  }

99  pass = true;

100  }

101  };

102  boost::phoenix::function<validate\_return\_allowed> validate\_return\_allowed\_f;

103

104  struct validate\_void\_return\_allowed {

105  template <typename T1, typename T2, typename T3>

106  struct result { typedef void type; };

107  void operator()(var\_origin origin,

108  bool& pass,

109  std::ostream& error\_msgs) const {

110  if (origin != void\_function\_argument\_origin

111  && origin != void\_function\_argument\_origin\_lp

112  && origin != void\_function\_argument\_origin\_rng) {

113  error\_msgs << "Void returns only allowed from function bodies of void return type."

114  << std::endl;

115  pass = false;

116  return;

117  }

118  pass = true;

119  }

120  };

121  boost::phoenix::function<validate\_void\_return\_allowed> validate\_void\_return\_allowed\_f;

122

123

124  struct validate\_assignment {

125  template <typename T1, typename T2, typename T3, typename T4>

126  struct result { typedef bool type; };

127

128  bool operator()(assignment& a,

129  const var\_origin& origin\_allowed,

130  variable\_map& vm,

131  std::ostream& error\_msgs) const {

132

133  // validate existence

134  std::string name = a.var\_dims\_.name\_;

135  if (!vm.exists(name)) {

136  error\_msgs << "unknown variable in assignment"

137  << "; lhs variable=" << a.var\_dims\_.name\_

138  << std::endl;

139  return false;

140  }

141

142  // validate origin

143  var\_origin lhs\_origin = vm.get\_origin(name);

144  if (lhs\_origin != local\_origin

145  && lhs\_origin != origin\_allowed) {

146  error\_msgs << "attempt to assign variable in wrong block."

147  << " left-hand-side variable origin=";

148  print\_var\_origin(error\_msgs,lhs\_origin);

149  error\_msgs << std::endl;

150  return false;

151  }

152

153  // enforce constancy of function args

154  if (lhs\_origin == function\_argument\_origin

155  || lhs\_origin == function\_argument\_origin\_lp

156  || lhs\_origin == function\_argument\_origin\_rng

157  || lhs\_origin == void\_function\_argument\_origin

158  || lhs\_origin == void\_function\_argument\_origin\_lp

159  || lhs\_origin == void\_function\_argument\_origin\_rng) {

160  error\_msgs << "Illegal to assign to function argument variables."

161  << std::endl

162  << "Use local variables instead."

163  << std::endl;

164  return false;

165  }

166

167

168  // validate types

169  a.var\_type\_ = vm.get(name);

170  size\_t lhs\_var\_num\_dims = a.var\_type\_.dims\_.size();

171  size\_t num\_index\_dims = a.var\_dims\_.dims\_.size();

172

173  expr\_type lhs\_type = infer\_type\_indexing(a.var\_type\_.base\_type\_,

174  lhs\_var\_num\_dims,

175  num\_index\_dims);

176

177  if (lhs\_type.is\_ill\_formed()) {

178  error\_msgs << "too many indexes for variable "

179  << "; variable name = " << name

180  << "; num dimensions given = " << num\_index\_dims

181  << "; variable array dimensions = " << lhs\_var\_num\_dims

182  << std::endl;

183  return false;

184  }

185

186  base\_expr\_type lhs\_base\_type = lhs\_type.base\_type\_;

187  base\_expr\_type rhs\_base\_type = a.expr\_.expression\_type().base\_type\_;

188  // allow int -> double promotion

189  bool types\_compatible

190  = lhs\_base\_type == rhs\_base\_type

191  || ( lhs\_base\_type == DOUBLE\_T && rhs\_base\_type == INT\_T );

192  if (!types\_compatible) {

193  error\_msgs << "base type mismatch in assignment"

194  << "; variable name = "

195  << a.var\_dims\_.name\_

196  << ", type = ";

197  write\_base\_expr\_type(error\_msgs,lhs\_base\_type);

198  error\_msgs << "; right-hand side type=";

199  write\_base\_expr\_type(error\_msgs,rhs\_base\_type);

200  error\_msgs << std::endl;

201  return false;

202  }

203  if (lhs\_type.num\_dims\_ != a.expr\_.expression\_type().num\_dims\_) {

204  error\_msgs << "dimension mismatch in assignment"

205  << "; variable name = "

206  << a.var\_dims\_.name\_

207  << ", num dimensions given = "

208  << lhs\_type.num\_dims\_

209  << "; right-hand side dimensions = "

210  << a.expr\_.expression\_type().num\_dims\_

211  << std::endl;

212  return false;

213  }

214  return true;

215  }

216  };

217  boost::phoenix::function<validate\_assignment> validate\_assignment\_f;

218

219  struct validate\_sample {

220  template <typename T1, typename T2, typename T3>

221  struct result { typedef bool type; };

222

223  bool is\_double\_return(const std::string& function\_name,

224  const std::vector<expr\_type>& arg\_types,

225  std::ostream& error\_msgs) const {

226  return function\_signatures::instance()

227  .get\_result\_type(function\_name,arg\_types,error\_msgs)

228  .is\_primitive\_double();

229  }

230  static bool is\_univariate(const expr\_type& et) {

231  return et.num\_dims\_ == 0

232  && ( et.base\_type\_ == INT\_T

233  || et.base\_type\_ == DOUBLE\_T );

234  }

235  bool operator()(const sample& s,

236  const variable\_map& var\_map,

237  std::ostream& error\_msgs) const {

238  std::vector<expr\_type> arg\_types;

239  arg\_types.push\_back(s.expr\_.expression\_type());

240  for (size\_t i = 0; i < s.dist\_.args\_.size(); ++i)

241  arg\_types.push\_back(s.dist\_.args\_[i].expression\_type());

242  std::string function\_name(s.dist\_.family\_);

243  function\_name += "\_log";

244  if (!is\_double\_return(function\_name,arg\_types,error\_msgs)) {

245  error\_msgs << "unknown distribution=" << s.dist\_.family\_ << std::endl;

246  return false;

247  }

248

249  if (function\_name == "lkj\_cov\_log") {

250  error\_msgs << "Warning: the lkj\_cov\_log() sampling distribution"

251  << " is deprecated. It will be removed in Stan 3."

252  << std::endl

253  << "Code LKJ covariance in terms of an lkj\_corr()"

254  << " distribution on a correlation matrix"

255  << " and independent lognormals on the scales."

256  << std::endl << std::endl;

257

258  }

259

260  // test for LHS not being purely a variable

261  if (has\_non\_param\_var(s.expr\_,var\_map)) {

262  // FIXME: really want to get line numbers in here too

263  error\_msgs << "Warning (non-fatal):"

264  << " Left-hand side of sampling statement (~) contains a non-linear"

265  << " transform of a parameter or local variable."

266  << std::endl

267  << " You must call increment\_log\_prob() with the log absolute determinant"

268  << " of the Jacobian of the transform."

269  << std::endl

270  << " Sampling Statement left-hand-side expression:"

271  << std::endl

272  << " ";

273  generate\_expression(s.expr\_,error\_msgs);

274  error\_msgs << " ~ ";

275  error\_msgs << function\_name << "(...)";

276  error\_msgs << std::endl;

277  }

278  // validate that variable and params are univariate if truncated

279  if (s.truncation\_.has\_low() || s.truncation\_.has\_high()) {

280  if (!is\_univariate(s.expr\_.expression\_type())) { // .num\_dims\_ > 0) {

281  error\_msgs << "Outcomes in truncated distributions must be univariate."

282  << std::endl

283  << " Found outcome expression: ";

284  generate\_expression(s.expr\_,error\_msgs);

285  error\_msgs << std::endl

286  << " with non-univariate type: "

287  << s.expr\_.expression\_type()

288  << std::endl;

289  return false;

290  }

291  for (size\_t i = 0; i < s.dist\_.args\_.size(); ++i)

292  if (!is\_univariate(s.dist\_.args\_[i].expression\_type())) { // .num\_dims\_ > 0) {

293  error\_msgs << "Parameters in truncated distributions must be univariate."

294  << std::endl

295  << " Found parameter expression: ";

296  generate\_expression(s.dist\_.args\_[i],error\_msgs);

297  error\_msgs << std::endl

298  << " with non-univariate type: "

299  << s.dist\_.args\_[i].expression\_type()

300  << std::endl;

301  return false;

302  }

303  }

304  if (s.truncation\_.has\_low()

305  && !is\_univariate(s.truncation\_.low\_.expression\_type())) {

306  error\_msgs << "Lower boundsin truncated distributions must be univariate."

307  << std::endl

308  << " Found lower bound expression: ";

309  generate\_expression(s.truncation\_.low\_,error\_msgs);

310  error\_msgs << std::endl

311  << " with non-univariate type: "

312  << s.truncation\_.low\_.expression\_type()

313  << std::endl;

314  return false;

315  }

316  if (s.truncation\_.has\_high()

317  && !is\_univariate(s.truncation\_.high\_.expression\_type())) {

318  error\_msgs << "Upper bounds in truncated distributions must be univariate."

319  << std::endl

320  << " Found upper bound expression: ";

321  generate\_expression(s.truncation\_.high\_,error\_msgs);

322  error\_msgs << std::endl

323  << " with non-univariate type: "

324  << s.truncation\_.high\_.expression\_type()

325  << std::endl;

326  return false;

327  }

328

329  if (s.truncation\_.has\_low()) {

330  std::vector<expr\_type> arg\_types\_trunc(arg\_types);

331  arg\_types\_trunc[0] = s.truncation\_.low\_.expression\_type();

332  std::string function\_name\_cdf(s.dist\_.family\_);

333  function\_name\_cdf += "\_cdf\_log";

334  if (!is\_double\_return(function\_name\_cdf,arg\_types\_trunc,error\_msgs)) {

335  error\_msgs << "lower truncation not defined for specified arguments to "

336  << s.dist\_.family\_ << std::endl;

337  return false;

338  }

339  if (!is\_double\_return(function\_name\_cdf,arg\_types,error\_msgs)) {

340  error\_msgs << "lower bound in truncation type does not match"

341  << " sampled variate in distribution's type"

342  << std::endl;

343  return false;

344  }

345  }

346  if (s.truncation\_.has\_high()) {

347  std::vector<expr\_type> arg\_types\_trunc(arg\_types);

348  arg\_types\_trunc[0] = s.truncation\_.high\_.expression\_type();

349  std::string function\_name\_cdf(s.dist\_.family\_);

350  function\_name\_cdf += "\_cdf\_log";

351  if (!is\_double\_return(function\_name\_cdf,arg\_types\_trunc,error\_msgs)) {

352  error\_msgs << "upper truncation not defined for specified arguments to "

353  << s.dist\_.family\_ << std::endl;

354  return false;

355  }

356  if (!is\_double\_return(function\_name\_cdf,arg\_types,error\_msgs)) {

357  error\_msgs << "upper bound in truncation type does not match"

358  << " sampled variate in distribution's type"

359  << std::endl;

360  return false;

361  }

362  }

363  return true;

364

365  }

366  };

367  boost::phoenix::function<validate\_sample> validate\_sample\_f;

368

369  struct expression\_as\_statement {

370  template <typename T1, typename T2, typename T3>

371  struct result { typedef void type; };

372  void operator()(bool& pass,

373  const stan::gm::expression& expr,

374  std::stringstream& error\_msgs) const {

375  if (expr.expression\_type() != VOID\_T) {

376  error\_msgs << "Illegal statement beginning with non-void expression parsed as"

377  << std::endl << " ";

378  generate\_expression(expr.expr\_,error\_msgs);

379  error\_msgs << std::endl

380  << "Not a legal assignment, sampling, or function statement. Note that"

381  << std::endl

382  << " \* Assignment statements only allow variables (with optional indexes) on the left;"

383  << std::endl

384  << " if you see an outer function logical\_lt (<) with negated (-) second argument,"

385  << std::endl

386  << " it indicates an assignment statement A <- B with illegal left"

387  << std::endl

388  << " side A parsed as expression (A < (-B))."

389  << std::endl

390  << " \* Sampling statements allow arbitrary value-denoting expressions on the left."

391  << std::endl

392  << " \* Functions used as statements must be declared to have void returns"

393  << std::endl << std::endl;

394  pass = false;

395  return;

396  }

397  pass = true;

398  }

399  };

400  boost::phoenix::function<expression\_as\_statement> expression\_as\_statement\_f;

401

402  struct unscope\_locals {

403  template <typename T1, typename T2>

404  struct result { typedef void type; };

405  void operator()(const std::vector<var\_decl>& var\_decls,

406  variable\_map& vm) const {

407  for (size\_t i = 0; i < var\_decls.size(); ++i)

408  vm.remove(var\_decls[i].name());

409  }

410  };

411  boost::phoenix::function<unscope\_locals> unscope\_locals\_f;

412

413  struct add\_while\_condition {

414  template <typename T1, typename T2, typename T3>

415  struct result { typedef bool type; };

416  bool operator()(while\_statement& ws,

417  const expression& e,

418  std::stringstream& error\_msgs) const {

419  if (!e.expression\_type().is\_primitive()) {

420  error\_msgs << "conditions in while statement must be primitive int or real;"

421  << " found type=" << e.expression\_type() << std::endl;

422  return false;

423  }

424  ws.condition\_ = e;

425  return true;

426  }

427  };

428  boost::phoenix::function<add\_while\_condition> add\_while\_condition\_f;

429

430  struct add\_while\_body {

431  template <typename T1, typename T2>

432  struct result { typedef void type; };

433  void operator()(while\_statement& ws,

434  const statement& s) const {

435  ws.body\_ = s;

436  }

437  };

438  boost::phoenix::function<add\_while\_body> add\_while\_body\_f;

439

440  struct add\_loop\_identifier {

441  template <typename T1, typename T2, typename T3, typename T4>

442  struct result { typedef bool type; };

443  bool operator()(const std::string& name,

444  std::string& name\_local,

445  variable\_map& vm,

446  std::stringstream& error\_msgs) const {

447  name\_local = name;

448  if (vm.exists(name)) {

449  error\_msgs << "ERROR: loop variable already declared."

450  << " variable name=\"" << name << "\"" << std::endl;

451  return false; // variable exists

452  }

453  vm.add(name,

454  base\_var\_decl(name,std::vector<expression>(),

455  INT\_T),

456  local\_origin); // loop var acts like local

457  return true;

458  }

459  };

460  boost::phoenix::function<add\_loop\_identifier> add\_loop\_identifier\_f;

461

462  struct remove\_loop\_identifier {

463  template <typename T1, typename T2>

464  struct result { typedef void type; };

465  void operator()(const std::string& name,

466  variable\_map& vm) const {

467  vm.remove(name);

468  }

469  };

470  boost::phoenix::function<remove\_loop\_identifier> remove\_loop\_identifier\_f;

471

472  struct validate\_int\_expr2 {

473  template <typename T1, typename T2, typename T3>

474  struct result { typedef void type; };

475

476  void operator()(const expression& expr,

477  bool& pass,

478  std::stringstream& error\_msgs) const {

479  if (!expr.expression\_type().is\_primitive\_int()) {

480  error\_msgs << "expression denoting integer required; found type="

481  << expr.expression\_type() << std::endl;

482  pass = false;

483  return;

484  }

485  pass = true;

486  return;

487  }

488  };

489  boost::phoenix::function<validate\_int\_expr2> validate\_int\_expr2\_f;

490

491  struct validate\_allow\_sample {

492  template <typename T1, typename T2, typename T3>

493  struct result { typedef void type; };

494

495  void operator()(const bool& allow\_sample,

496  bool& pass,

497  std::stringstream& error\_msgs) const {

498  if (!allow\_sample) {

499  error\_msgs << "sampling only allowed in model."

500  << std::endl;

501  pass = false;

502  return;

503  }

504  pass = true;

505  return;

506  }

507  };

508  boost::phoenix::function<validate\_allow\_sample> validate\_allow\_sample\_f;

509

510  struct validate\_non\_void\_expression {

511  template <typename T1, typename T2, typename T3>

512  struct result { typedef void type; };

513

514  void operator()(const expression& e,

515  bool& pass,

516  std::ostream& error\_msgs) const {

517  pass = !e.expression\_type().is\_void();

518  if (!pass) {

519  error\_msgs << "attempt to increment log prob with void expression" << std::endl;

520  }

521  }

522  };

523  boost::phoenix::function<validate\_non\_void\_expression> validate\_non\_void\_expression\_f;

524

525

526  template <typename Iterator>

527  statement\_grammar<Iterator>::statement\_grammar(variable\_map& var\_map,

528  std::stringstream& error\_msgs)

529  : statement\_grammar::base\_type(statement\_r),

530  var\_map\_(var\_map),

531  error\_msgs\_(error\_msgs),

532  expression\_g(var\_map,error\_msgs),

533  var\_decls\_g(var\_map,error\_msgs),

534  statement\_2\_g(var\_map,error\_msgs,\*this)

535  {

536  using boost::spirit::qi::\_1;

537  using boost::spirit::qi::char\_;

538  using boost::spirit::qi::eps;

539  using boost::spirit::qi::lexeme;

540  using boost::spirit::qi::lit;

541  using boost::spirit::qi::no\_skip;

542  using boost::spirit::qi::\_pass;

543  using boost::spirit::qi::\_val;

544

545  using boost::spirit::qi::labels::\_a;

546  using boost::spirit::qi::labels::\_r1;

547  using boost::spirit::qi::labels::\_r2;

548  using boost::spirit::qi::labels::\_r3;

549

550  // inherited features

551  // \_r1 true if sample\_r allowed

552  // \_r2 source of variables allowed for assignments

553  // \_r3 true if return\_r allowed

554  statement\_r.name("statement");

555  statement\_r

556  %= no\_op\_statement\_r // key ";"

557  | statement\_seq\_r(\_r1,\_r2,\_r3) // key "{"

558  | increment\_log\_prob\_statement\_r(\_r1,\_r2) // key "increment\_log\_prob"

559  | for\_statement\_r(\_r1,\_r2,\_r3) // key "for"

560  | while\_statement\_r(\_r1,\_r2,\_r3) // key "while"

561  | statement\_2\_g(\_r1,\_r2,\_r3) // key "if"

562  | print\_statement\_r(\_r2) // key "print"

563  | reject\_statement\_r(\_r2) // key "reject"

564  | return\_statement\_r(\_r2) // key "return"

565  | void\_return\_statement\_r(\_r2) // key "return"

566  | assignment\_r(\_r2) // lvalue "<-"

567  | sample\_r(\_r1,\_r2) // expression "~"

568  | expression\_g(\_r2) // expression

569  [expression\_as\_statement\_f(\_pass,\_1,boost::phoenix::ref(error\_msgs\_))]

570  ;

571

572  // \_r1, \_r2, \_r3 same as statement\_r

573  statement\_seq\_r.name("sequence of statements");

574  statement\_seq\_r

575  %= lit('{')

576  > local\_var\_decls\_r[\_a = \_1]

577  > \*statement\_r(\_r1,\_r2,\_r3)

578  > lit('}')

579  > eps[unscope\_locals\_f(\_a,boost::phoenix::ref(var\_map\_))]

580  ;

581

582  local\_var\_decls\_r

583  %= var\_decls\_g(false,local\_origin); // - constants

584

585  // inherited \_r1 = true if samples allowed as statements

586  increment\_log\_prob\_statement\_r.name("increment log prob statement");

587  increment\_log\_prob\_statement\_r

588  %= lit("increment\_log\_prob")

589  > eps[ validate\_allow\_sample\_f(\_r1,\_pass,

590  boost::phoenix::ref(error\_msgs\_)) ]

591  > lit('(')

592  > expression\_g(\_r2) [ validate\_non\_void\_expression\_f(\_1,\_pass,

593  boost::phoenix::ref(error\_msgs\_)) ]

594  > lit(')')

595  > lit(';')

596  ;

597

598  // \_r1, \_r2, \_r3 same as statement\_r

599  while\_statement\_r.name("while statement");

600  while\_statement\_r

601  = lit("while")

602  > lit('(')

603  > expression\_g(\_r2)

604  [\_pass = add\_while\_condition\_f(\_val,\_1,

605  boost::phoenix::ref(error\_msgs\_))]

606  > lit(')')

607  > statement\_r(\_r1,\_r2,\_r3)

608  [add\_while\_body\_f(\_val,\_1)]

609  ;

610

611

612  // \_r1, \_r2, \_r3 same as statement\_r

613  for\_statement\_r.name("for statement");

614  for\_statement\_r

615  %= lit("for")

616  > lit('(')

617  > identifier\_r [\_pass

618  = add\_loop\_identifier\_f(\_1,\_a,

619  boost::phoenix::ref(var\_map\_),

620  boost::phoenix::ref(error\_msgs\_))]

621  > lit("in")

622  > range\_r(\_r2)

623  > lit(')')

624  > statement\_r(\_r1,\_r2,\_r3)

625  > eps

626  [remove\_loop\_identifier\_f(\_a,boost::phoenix::ref(var\_map\_))];

627  ;

628

629  print\_statement\_r.name("print statement");

630  print\_statement\_r

631  %= lit("print")

632  > lit('(')

633  > (printable\_r(\_r1) % ',')

634  > lit(')');

635

636  // reject

637  reject\_statement\_r.name("reject statement");

638  reject\_statement\_r

639  %= lit("reject")

640  > lit('(')

641  > (printable\_r(\_r1) % ',')

642  > lit(')');

643

644  printable\_r.name("printable");

645  printable\_r

646  %= printable\_string\_r

647  | expression\_g(\_r1);

648

649  printable\_string\_r.name("printable quoted string");

650  printable\_string\_r

651  %= lit('"')

652  > no\_skip[\*char\_("a-zA-Z0-9/~!@#$%^&\*()`\_+-={}|[]:;'<>?,./ ")]

653  > lit('"');

654

655  identifier\_r.name("identifier");

656  identifier\_r

657  %= (lexeme[char\_("a-zA-Z")

658  >> \*char\_("a-zA-Z0-9\_.")]);

659

660  range\_r.name("range expression pair, colon");

661  range\_r

662  %= expression\_g(\_r1)

663  [validate\_int\_expr2\_f(\_1,\_pass,boost::phoenix::ref(error\_msgs\_))]

664  >> lit(':')

665  >> expression\_g(\_r1)

666  [validate\_int\_expr2\_f(\_1,\_pass,boost::phoenix::ref(error\_msgs\_))];

667

668  assignment\_r.name("variable assignment by expression");

669  assignment\_r

670  %= ( var\_lhs\_r(\_r1)

671  >> lit("<-") )

672  > expression\_g(\_r1)

673  > lit(';')

674  [\_pass = validate\_assignment\_f(\_val,\_r1,boost::phoenix::ref(var\_map\_),

675  boost::phoenix::ref(error\_msgs\_))]

676  ;

677

678  var\_lhs\_r.name("variable and array dimensions");

679  var\_lhs\_r

680  %= identifier\_r

681  >> opt\_dims\_r(\_r1);

682

683  opt\_dims\_r.name("array dimensions (optional)");

684  opt\_dims\_r

685  %= \* dims\_r(\_r1);

686

687  dims\_r.name("array dimensions");

688  dims\_r

689  %= lit('[')

690  > (expression\_g(\_r1)

691  [validate\_int\_expr2\_f(\_1,\_pass,boost::phoenix::ref(error\_msgs\_))]

692  % ',')

693  > lit(']')

694  ;

695

696  // inherited \_r1 = true if samples allowed as statements

697  sample\_r.name("distribution of expression");

698  sample\_r

699  %= ( expression\_g(\_r2)

700  >> lit('~') )

701  > eps

702  [validate\_allow\_sample\_f(\_r1,\_pass,

703  boost::phoenix::ref(error\_msgs\_))]

704  > distribution\_r(\_r2)

705  > -truncation\_range\_r(\_r2)

706  > lit(';')

707  > eps

708  [\_pass = validate\_sample\_f(\_val,

709  boost::phoenix::ref(var\_map\_),

710  boost::phoenix::ref(error\_msgs\_))]

711  ;

712

713  distribution\_r.name("distribution and parameters");

714  distribution\_r

715  %= ( identifier\_r

716  >> lit('(')

717  >> -(expression\_g(\_r1) % ',') )

718  > lit(')')

719  ;

720

721  truncation\_range\_r.name("range pair");

722  truncation\_range\_r

723  %= lit('T')

724  > lit('[')

725  > -expression\_g(\_r1)

726  > lit(',')

727  > -expression\_g(\_r1)

728  > lit(']')

729  ;

730

731  // \_r1 = allow sampling, \_r2 = var origin

732  return\_statement\_r.name("return statement");

733  return\_statement\_r

734  %= lit("return")

735  >> expression\_g(\_r1)

736  >> lit(';') [ validate\_return\_allowed\_f(\_r1,\_pass,

737  boost::phoenix::ref(error\_msgs\_)) ]

738  ;

739

740  // \_r1 = var origin

741  void\_return\_statement\_r.name("void return statement");

742  void\_return\_statement\_r

743  = lit("return")[\_val = expression()]

744  >> lit(';') [ validate\_void\_return\_allowed\_f(\_r1,\_pass,

745  boost::phoenix::ref(error\_msgs\_)) ]

746  ;

747

748  no\_op\_statement\_r.name("no op statement");

749  no\_op\_statement\_r

750  %= lit(';') [\_val = no\_op\_statement()]; // ok to re-use instance

751

752  using boost::spirit::qi::on\_error;

753  using boost::spirit::qi::fail;

754  using boost::spirit::qi::rethrow;

755  using namespace boost::spirit::qi::labels;

756

757  }

758

759  }

760 }

761 #endif

stan::gm::distribution::family\_

std::string family\_

**Definition:** ast.hpp:195

stan::gm::expression\_as\_statement::result

**Definition:** statement\_grammar\_def.hpp:371

stan::gm::validate\_non\_void\_expression::result

**Definition:** statement\_grammar\_def.hpp:512

stan::gm::add\_while\_body::operator()

void operator()(while\_statement &ws, const statement &s) const

**Definition:** statement\_grammar\_def.hpp:433

stan::gm::variable\_dims

**Definition:** ast.hpp:288

stan::gm::validate\_assignment\_f

boost::phoenix::function< validate\_assignment > validate\_assignment\_f

**Definition:** statement\_grammar\_def.hpp:217

stan::gm::statement

**Definition:** ast.hpp:634

stan::gm::generate\_expression

void generate\_expression(const expression &e, std::ostream &o)

**Definition:** generator.hpp:249

stan::gm::statement\_grammar::printable\_r

boost::spirit::qi::rule< Iterator, printable(var\_origin), whitespace\_grammar< Iterator > > printable\_r

**Definition:** statement\_grammar.hpp:110

stan::gm::validate\_non\_void\_expression::operator()

void operator()(const expression &e, bool &pass, std::ostream &error\_msgs) const

**Definition:** statement\_grammar\_def.hpp:514

stan::gm::validate\_allow\_sample::operator()

void operator()(const bool &allow\_sample, bool &pass, std::stringstream &error\_msgs) const

**Definition:** statement\_grammar\_def.hpp:495

stan::gm::validate\_void\_return\_allowed::result::type

void type

**Definition:** statement\_grammar\_def.hpp:106

stan::gm::base\_var\_decl::dims\_

std::vector< expression > dims\_

**Definition:** ast.hpp:430

stan::gm::expr\_type::base\_type\_

base\_expr\_type base\_type\_

**Definition:** ast.hpp:75

stan::gm::unscope\_locals::operator()

void operator()(const std::vector< var\_decl > &var\_decls, variable\_map &vm) const

**Definition:** statement\_grammar\_def.hpp:405

stan::gm::assignment::var\_dims\_

variable\_dims var\_dims\_

**Definition:** ast.hpp:829

stan::gm::statement\_grammar::range\_r

boost::spirit::qi::rule< Iterator, range(var\_origin), whitespace\_grammar< Iterator > > range\_r

**Definition:** statement\_grammar.hpp:141

stan::gm::variable\_map::add

void add(const std::string &name, const base\_var\_decl &base\_decl, const var\_origin &vo)

**Definition:** ast\_def.cpp:1007

stan::gm::unscope\_locals::result::type

void type

**Definition:** statement\_grammar\_def.hpp:404

stan::gm::DOUBLE\_T

const int DOUBLE\_T

**Definition:** ast.hpp:66

stan::gm::remove\_loop\_identifier::result::type

void type

**Definition:** statement\_grammar\_def.hpp:464

stan::gm::function\_signatures::instance

static function\_signatures & instance()

**Definition:** ast\_def.cpp:143

stan::gm::statement\_grammar

**Definition:** statement\_2\_grammar.hpp:19

stan::gm::validate\_sample

**Definition:** statement\_grammar\_def.hpp:219

stan::gm::local\_origin

const int local\_origin

**Definition:** ast.hpp:418

stan::gm::distribution

**Definition:** ast.hpp:194

stan::gm::assignment::expr\_

expression expr\_

**Definition:** ast.hpp:830

stan::gm::statement\_grammar::for\_statement\_r

boost::spirit::qi::rule< Iterator, boost::spirit::qi::locals< std::string >, for\_statement(bool, var\_origin, bool), whitespace\_grammar< Iterator > > for\_statement\_r

**Definition:** statement\_grammar.hpp:74

stan::gm::validate\_allow\_sample\_f

boost::phoenix::function< validate\_allow\_sample > validate\_allow\_sample\_f

**Definition:** statement\_grammar\_def.hpp:508

stan::gm::statements

**Definition:** ast.hpp:185

stan::gm::range

**Definition:** ast.hpp:401

stan::gm::assignment

**Definition:** ast.hpp:828

stan::gm::validate\_sample::operator()

bool operator()(const sample &s, const variable\_map &var\_map, std::ostream &error\_msgs) const

**Definition:** statement\_grammar\_def.hpp:235

expression\_grammar.hpp

stan::gm::expr\_type::is\_primitive\_int

bool is\_primitive\_int() const

**Definition:** ast\_def.cpp:97

stan::gm::statement\_grammar::local\_var\_decls\_r

boost::spirit::qi::rule< Iterator, std::vector< var\_decl >), whitespace\_grammar< Iterator > > local\_var\_decls\_r

**Definition:** statement\_grammar.hpp:126

stan::gm::statement\_grammar::identifier\_r

boost::spirit::qi::rule< Iterator, std::string(), whitespace\_grammar< Iterator > > identifier\_r

**Definition:** statement\_grammar.hpp:121

stan::gm::range::has\_low

bool has\_low() const

**Definition:** ast\_def.cpp:937

stan::gm::range::high\_

expression high\_

**Definition:** ast.hpp:403

stan::gm::remove\_loop\_identifier

**Definition:** statement\_grammar\_def.hpp:462

stan::gm::expr\_type::is\_primitive\_double

bool is\_primitive\_double() const

**Definition:** ast\_def.cpp:101

stan::gm::expression\_as\_statement::operator()

void operator()(bool &pass, const stan::gm::expression &expr, std::stringstream &error\_msgs) const

**Definition:** statement\_grammar\_def.hpp:372

stan::gm::print\_var\_origin

void print\_var\_origin(std::ostream &o, const var\_origin &vo)

**Definition:** ast\_def.cpp:944

stan::gm::function\_argument\_origin

const int function\_argument\_origin

**Definition:** ast.hpp:419

stan::gm::validate\_allow\_sample::result

**Definition:** statement\_grammar\_def.hpp:493

stan::gm::no\_op\_statement

**Definition:** ast.hpp:763

stan::gm::statement\_grammar::reject\_statement\_r

boost::spirit::qi::rule< Iterator, reject\_statement(var\_origin), whitespace\_grammar< Iterator > > reject\_statement\_r

**Definition:** statement\_grammar.hpp:91

statement\_grammar.hpp

stan::gm::unscope\_locals\_f

boost::phoenix::function< unscope\_locals > unscope\_locals\_f

**Definition:** statement\_grammar\_def.hpp:411

stan::gm::statement\_grammar::truncation\_range\_r

boost::spirit::qi::rule< Iterator, range(var\_origin), whitespace\_grammar< Iterator > > truncation\_range\_r

**Definition:** statement\_grammar.hpp:162

stan::gm::statement\_grammar::printable\_string\_r

boost::spirit::qi::rule< Iterator, std::string(), whitespace\_grammar< Iterator > > printable\_string\_r

**Definition:** statement\_grammar.hpp:115

stan::gm::validate\_sample::result::type

bool type

**Definition:** statement\_grammar\_def.hpp:221

stan::gm::add\_while\_condition::operator()

bool operator()(while\_statement &ws, const expression &e, std::stringstream &error\_msgs) const

**Definition:** statement\_grammar\_def.hpp:416

stan::gm::validate\_assignment::result::type

bool type

**Definition:** statement\_grammar\_def.hpp:126

stan::gm::void\_function\_argument\_origin\_rng

const int void\_function\_argument\_origin\_rng

**Definition:** ast.hpp:424

stan::gm::statement\_grammar::statement\_2\_g

statement\_2\_grammar< Iterator > statement\_2\_g

**Definition:** statement\_grammar.hpp:39

stan::gm::function\_argument\_origin\_rng

const int function\_argument\_origin\_rng

**Definition:** ast.hpp:421

stan::gm::statement\_grammar::statement\_r

boost::spirit::qi::rule< Iterator, statement(bool, var\_origin, bool), whitespace\_grammar< Iterator > > statement\_r

**Definition:** statement\_grammar.hpp:151

stan::gm::remove\_loop\_identifier::result

**Definition:** statement\_grammar\_def.hpp:464

stan::gm::for\_statement

**Definition:** ast.hpp:718

stan::gm::increment\_log\_prob\_statement

**Definition:** ast.hpp:712

stan::gm::add\_while\_body

**Definition:** statement\_grammar\_def.hpp:430

stan::gm::print\_statement

**Definition:** ast.hpp:745

stan::gm::sample::expr\_

expression expr\_

**Definition:** ast.hpp:819

stan::gm::base\_var\_decl::base\_type\_

base\_expr\_type base\_type\_

**Definition:** ast.hpp:431

stan::gm::statement\_grammar::statement\_seq\_r

boost::spirit::qi::rule< Iterator, boost::spirit::qi::locals< std::vector< var\_decl > >, statements(bool, var\_origin, bool), whitespace\_grammar< Iterator > > statement\_seq\_r

**Definition:** statement\_grammar.hpp:157

stan::gm::expression::expr\_

expression\_t expr\_

**Definition:** ast.hpp:252

stan::gm::while\_statement::body\_

statement body\_

**Definition:** ast.hpp:739

stan::gm::add\_loop\_identifier

**Definition:** statement\_grammar\_def.hpp:440

stan::gm::expr\_type::num\_dims\_

size\_t num\_dims\_

**Definition:** ast.hpp:76

stan::gm::has\_non\_param\_var

bool has\_non\_param\_var(const expression &e, const variable\_map &var\_map)

**Definition:** ast\_def.cpp:748

stan::gm::statement\_grammar::error\_msgs\_

std::stringstream & error\_msgs\_

**Definition:** statement\_grammar.hpp:34

stan::gm::expression\_as\_statement::result::type

void type

**Definition:** statement\_grammar\_def.hpp:371

stan::gm::base\_var\_decl

**Definition:** ast.hpp:428

stan::gm::while\_statement::condition\_

expression condition\_

**Definition:** ast.hpp:738

stan::gm::validate\_sample::is\_univariate

static bool is\_univariate(const expr\_type &et)

**Definition:** statement\_grammar\_def.hpp:230

stan::gm::statement\_grammar::print\_statement\_r

boost::spirit::qi::rule< Iterator, print\_statement(var\_origin), whitespace\_grammar< Iterator > > print\_statement\_r

**Definition:** statement\_grammar.hpp:86

stan::gm::validate\_non\_void\_expression

**Definition:** statement\_grammar\_def.hpp:510

stan::gm::variable\_map

**Definition:** ast.hpp:439

stan::gm::range::low\_

expression low\_

**Definition:** ast.hpp:402

stan::gm::expr\_type

**Definition:** ast.hpp:74

stan::gm::add\_while\_condition::result

**Definition:** statement\_grammar\_def.hpp:415

stan::gm::variable\_dims::dims\_

std::vector< expression > dims\_

**Definition:** ast.hpp:290

stan::gm::validate\_void\_return\_allowed::result

**Definition:** statement\_grammar\_def.hpp:106

stan::gm::void\_function\_argument\_origin\_lp

const int void\_function\_argument\_origin\_lp

**Definition:** ast.hpp:423

stan::gm::variable\_dims::name\_

std::string name\_

**Definition:** ast.hpp:289

stan::gm::statement\_grammar::while\_statement\_r

boost::spirit::qi::rule< Iterator, while\_statement(bool, var\_origin, bool), whitespace\_grammar< Iterator > > while\_statement\_r

**Definition:** statement\_grammar.hpp:80

stan::gm::add\_while\_body::result::type

void type

**Definition:** statement\_grammar\_def.hpp:432

stan::gm::add\_loop\_identifier::result

**Definition:** statement\_grammar\_def.hpp:442

stan::gm::validate\_assignment::result

**Definition:** statement\_grammar\_def.hpp:126

stan::gm::validate\_return\_allowed::operator()

void operator()(var\_origin origin, bool &pass, std::ostream &error\_msgs) const

**Definition:** statement\_grammar\_def.hpp:89

stan::gm::expression

**Definition:** ast.hpp:216

stan::gm::validate\_return\_allowed::result

**Definition:** statement\_grammar\_def.hpp:88

stan::gm::validate\_return\_allowed::result::type

void type

**Definition:** statement\_grammar\_def.hpp:88

stan::gm::unscope\_locals

**Definition:** statement\_grammar\_def.hpp:402

var\_decls\_grammar.hpp

stan::gm::void\_function\_argument\_origin

const int void\_function\_argument\_origin

**Definition:** ast.hpp:422

stan::gm::statement\_grammar::var\_map\_

variable\_map & var\_map\_

**Definition:** statement\_grammar.hpp:33

stan::gm::statement\_grammar::var\_decls\_g

var\_decls\_grammar< Iterator > var\_decls\_g

**Definition:** statement\_grammar.hpp:38

stan::gm::statement\_grammar::expression\_g

expression\_grammar< Iterator > expression\_g

**Definition:** statement\_grammar.hpp:37

stan::gm::sample

**Definition:** ast.hpp:818

stan::gm::add\_while\_condition

**Definition:** statement\_grammar\_def.hpp:413

stan::gm::validate\_sample::result

**Definition:** statement\_grammar\_def.hpp:221

stan::gm::remove\_loop\_identifier::operator()

void operator()(const std::string &name, variable\_map &vm) const

**Definition:** statement\_grammar\_def.hpp:465

stan::gm::validate\_int\_expr2

**Definition:** statement\_grammar\_def.hpp:472

stan::gm::reject\_statement

**Definition:** ast.hpp:751

stan::gm::statement\_grammar::var\_lhs\_r

boost::spirit::qi::rule< Iterator, variable\_dims(var\_origin), whitespace\_grammar< Iterator > > var\_lhs\_r

**Definition:** statement\_grammar.hpp:167

stan::gm::add\_while\_body::result

**Definition:** statement\_grammar\_def.hpp:432

ast.hpp

stan::gm::add\_while\_condition::result::type

bool type

**Definition:** statement\_grammar\_def.hpp:415

stan::gm::validate\_void\_return\_allowed::operator()

void operator()(var\_origin origin, bool &pass, std::ostream &error\_msgs) const

**Definition:** statement\_grammar\_def.hpp:107

stan::gm::statement\_grammar::void\_return\_statement\_r

boost::spirit::qi::rule< Iterator, return\_statement(var\_origin), whitespace\_grammar< Iterator > > void\_return\_statement\_r

**Definition:** statement\_grammar.hpp:102

stan::gm::statement\_grammar::dims\_r

boost::spirit::qi::rule< Iterator, std::vector< expression >var\_origin), whitespace\_grammar< Iterator > > dims\_r

**Definition:** statement\_grammar.hpp:56

stan::gm::base\_expr\_type

int base\_expr\_type

**Definition:** ast.hpp:60

stan::gm::add\_while\_condition\_f

boost::phoenix::function< add\_while\_condition > add\_while\_condition\_f

**Definition:** statement\_grammar\_def.hpp:428

stan::gm::validate\_allow\_sample::result::type

void type

**Definition:** statement\_grammar\_def.hpp:493

stan::gm::statement\_grammar::no\_op\_statement\_r

boost::spirit::qi::rule< Iterator, no\_op\_statement(), whitespace\_grammar< Iterator > > no\_op\_statement\_r

**Definition:** statement\_grammar.hpp:131

stan::math::e

double e()

Return the base of the natural logarithm.

**Definition:** constants.hpp:86

stan::gm::add\_loop\_identifier::operator()

bool operator()(const std::string &name, std::string &name\_local, variable\_map &vm, std::stringstream &error\_msgs) const

**Definition:** statement\_grammar\_def.hpp:443

stan::gm::sample::truncation\_

range truncation\_

**Definition:** ast.hpp:821

stan::gm::validate\_assignment

**Definition:** statement\_grammar\_def.hpp:124

stan::gm::add\_loop\_identifier::result::type

bool type

**Definition:** statement\_grammar\_def.hpp:442

stan::gm::expr\_type::is\_ill\_formed

bool is\_ill\_formed() const

**Definition:** ast\_def.cpp:105

stan::gm::variable\_map::remove

void remove(const std::string &name)

**Definition:** ast\_def.cpp:1012

stan::gm::validate\_int\_expr2::operator()

void operator()(const expression &expr, bool &pass, std::stringstream &error\_msgs) const

**Definition:** statement\_grammar\_def.hpp:476

stan::gm::statement\_grammar::increment\_log\_prob\_statement\_r

boost::spirit::qi::rule< Iterator, increment\_log\_prob\_statement(bool, var\_origin), whitespace\_grammar< Iterator > > increment\_log\_prob\_statement\_r

**Definition:** statement\_grammar.hpp:68

stan::gm::statement\_grammar::assignment\_r

boost::spirit::qi::rule< Iterator, assignment(var\_origin), whitespace\_grammar< Iterator > > assignment\_r

**Definition:** statement\_grammar.hpp:45

stan::gm::range::has\_high

bool has\_high() const

**Definition:** ast\_def.cpp:940

whitespace\_grammar.hpp

stan::gm::infer\_type\_indexing

expr\_type infer\_type\_indexing(const base\_expr\_type &expr\_base\_type, size\_t num\_expr\_dims, size\_t num\_index\_dims)

**Definition:** ast\_def.cpp:873

stan::gm::validate\_int\_expr2\_f

boost::phoenix::function< validate\_int\_expr2 > validate\_int\_expr2\_f

**Definition:** statement\_grammar\_def.hpp:489

stan::gm::validate\_sample\_f

boost::phoenix::function< validate\_sample > validate\_sample\_f

**Definition:** statement\_grammar\_def.hpp:367

stan::gm::function\_argument\_origin\_lp

const int function\_argument\_origin\_lp

**Definition:** ast.hpp:420

common\_adaptors\_def.hpp

stan::gm::INT\_T

const int INT\_T

**Definition:** ast.hpp:65

stan::gm::statement\_grammar::sample\_r

boost::spirit::qi::rule< Iterator, sample(bool, var\_origin), whitespace\_grammar< Iterator > > sample\_r

**Definition:** statement\_grammar.hpp:146

stan::gm::assignment::var\_type\_

base\_var\_decl var\_type\_

**Definition:** ast.hpp:831

stan::gm::validate\_non\_void\_expression\_f

boost::phoenix::function< validate\_non\_void\_expression > validate\_non\_void\_expression\_f

**Definition:** statement\_grammar\_def.hpp:523

stan::gm::validate\_assignment::operator()

bool operator()(assignment &a, const var\_origin &origin\_allowed, variable\_map &vm, std::ostream &error\_msgs) const

**Definition:** statement\_grammar\_def.hpp:128

stan::gm::return\_statement

**Definition:** ast.hpp:757

stan::gm::distribution::args\_

std::vector< expression > args\_

**Definition:** ast.hpp:196

stan::gm::unscope\_locals::result

**Definition:** statement\_grammar\_def.hpp:404

stan::gm::statement\_grammar::opt\_dims\_r

boost::spirit::qi::rule< Iterator, std::vector< expression >var\_origin), whitespace\_grammar< Iterator > > opt\_dims\_r

**Definition:** statement\_grammar.hpp:136

stan::gm::add\_while\_body\_f

boost::phoenix::function< add\_while\_body > add\_while\_body\_f

**Definition:** statement\_grammar\_def.hpp:438

stan::gm::validate\_non\_void\_expression::result::type

void type

**Definition:** statement\_grammar\_def.hpp:512

BOOST\_FUSION\_ADAPT\_STRUCT

BOOST\_FUSION\_ADAPT\_STRUCT(stan::gm::assignment,(stan::gm::variable\_dims, var\_dims\_)(stan::gm::expression, expr\_))

stan::gm::sample::dist\_

distribution dist\_

**Definition:** ast.hpp:820

stan::gm::validate\_void\_return\_allowed

**Definition:** statement\_grammar\_def.hpp:104

stan::gm::function\_signatures::get\_result\_type

expr\_type get\_result\_type(const std::string &name, const std::vector< expr\_type > &args, std::ostream &error\_msgs)

**Definition:** ast\_def.cpp:329

stan::gm::remove\_loop\_identifier\_f

boost::phoenix::function< remove\_loop\_identifier > remove\_loop\_identifier\_f

**Definition:** statement\_grammar\_def.hpp:470

stan::gm::validate\_void\_return\_allowed\_f

boost::phoenix::function< validate\_void\_return\_allowed > validate\_void\_return\_allowed\_f

**Definition:** statement\_grammar\_def.hpp:121

stan::gm::while\_statement

**Definition:** ast.hpp:737

stan::gm::variable\_map::exists

bool exists(const std::string &name) const

**Definition:** ast\_def.cpp:988

stan::gm::write\_base\_expr\_type

std::ostream & write\_base\_expr\_type(std::ostream &o, base\_expr\_type type)

**Definition:** ast\_def.cpp:23

stan::gm::validate\_return\_allowed

**Definition:** statement\_grammar\_def.hpp:86

stan::gm::expression::expression\_type

expr\_type expression\_type() const

**Definition:** ast\_def.cpp:570

stan::gm::expression\_as\_statement\_f

boost::phoenix::function< expression\_as\_statement > expression\_as\_statement\_f

**Definition:** statement\_grammar\_def.hpp:400

stan::gm::variable\_map::get

base\_var\_decl get(const std::string &name) const

**Definition:** ast\_def.cpp:991

stan::gm::statement\_grammar::distribution\_r

boost::spirit::qi::rule< Iterator, distribution(var\_origin), whitespace\_grammar< Iterator > > distribution\_r

**Definition:** statement\_grammar.hpp:62

stan::gm::validate\_allow\_sample

**Definition:** statement\_grammar\_def.hpp:491

stan::gm::add\_loop\_identifier\_f

boost::phoenix::function< add\_loop\_identifier > add\_loop\_identifier\_f

**Definition:** statement\_grammar\_def.hpp:460

stan::gm::validate\_sample::is\_double\_return

bool is\_double\_return(const std::string &function\_name, const std::vector< expr\_type > &arg\_types, std::ostream &error\_msgs) const

**Definition:** statement\_grammar\_def.hpp:223

stan::gm::var\_origin

int var\_origin

**Definition:** ast.hpp:411

stan::gm::expr\_type::is\_primitive

bool is\_primitive() const

**Definition:** ast\_def.cpp:93

stan::gm::validate\_return\_allowed\_f

boost::phoenix::function< validate\_return\_allowed > validate\_return\_allowed\_f

**Definition:** statement\_grammar\_def.hpp:102

stan::gm::validate\_int\_expr2::result::type

void type

**Definition:** statement\_grammar\_def.hpp:474

stan::gm::variable\_map::get\_origin

var\_origin get\_origin(const std::string &name) const

**Definition:** ast\_def.cpp:1002

stan::gm::expr\_type::is\_void

bool is\_void() const

**Definition:** ast\_def.cpp:108

stan::gm::VOID\_T

const int VOID\_T

**Definition:** ast.hpp:64

stan::gm::validate\_int\_expr2::result

**Definition:** statement\_grammar\_def.hpp:474

stan::gm::statement\_grammar::return\_statement\_r

boost::spirit::qi::rule< Iterator, return\_statement(var\_origin), whitespace\_grammar< Iterator > > return\_statement\_r

**Definition:** statement\_grammar.hpp:97

stan::gm::expression\_as\_statement

**Definition:** statement\_grammar\_def.hpp:369

stan::gm::statement\_grammar::statement\_grammar

statement\_grammar(variable\_map &var\_map, std::stringstream &error\_msgs)

**Definition:** statement\_grammar\_def.hpp:527


---

[ Stan Home Page ]

*© 2011–2014,
Stan Development Team.*
